# Monitoring what is real: The effects of modality and action on accuracy and type of reality monitoring error

**DOI:** 10.1016/j.cortex.2016.06.018

**Published:** 2017-02

**Authors:** Jane R. Garrison, Rebecca Bond, Emma Gibbard, Marcia K. Johnson, Jon S. Simons

**Affiliations:** a Department of Psychology, University of Cambridge, UK; b Behavioural and Clinical Neuroscience Institute, University of Cambridge, UK; c Department of Psychology, Yale University, New Haven, CT, USA

**Keywords:** Schizophrenia, Confabulation, Hallucinations, Delusions, Reality monitoring

## Abstract

Reality monitoring refers to processes involved in distinguishing internally generated information from information presented in the external world, an activity thought to be based, in part, on assessment of activated features such as the amount and type of cognitive operations and perceptual content. Impairment in reality monitoring has been implicated in symptoms of mental illness and associated more widely with the occurrence of anomalous perceptions as well as false memories and beliefs. In the present experiment, the cognitive mechanisms of reality monitoring were probed in healthy individuals using a task that investigated the effects of stimulus modality (auditory *vs* visual) and the type of action undertaken during encoding (thought *vs* speech) on subsequent source memory. There was reduced source accuracy for auditory stimuli compared with visual, and when encoding was accompanied by thought as opposed to speech, and a greater rate of externalization than internalization errors that was stable across factors. Interpreted within the source monitoring framework (Johnson, Hashtroudi, & Lindsay, 1993), the results are consistent with the greater prevalence of clinically observed auditory than visual reality discrimination failures. The significance of these findings is discussed in light of theories of hallucinations, delusions and confabulation.

## 1 Introduction

The source monitoring framework (SMF) proposes that memories do not contain labels or tags that directly specify their source, but instead that the origin of memories is inferred, for example, from characteristic features (Johnson, Hashtroudi, & Lindsay, 1993). Such features might comprise: (i) contextual attributes such as spatial or temporal detail, (ii) sensory attributes such as colour or pitch, (iii) semantic information and emotional qualities, and (iv) internal cognitive operations such as those involved in reasoning or thinking about events. For example, if memory for a news story contains auditory but no visual features, its origin might be attributed to the radio rather than TV.

If the source monitoring judgement relates to the internal or external origin of the memory (that is, whether an event was imagined or really did occur), the attribution process is referred to as *reality monitoring* (Johnson & Raye, 1981). Memory traces of perceived and imagined events are different on average, with greater cognitive operations content for self-generated information and greater sensory and contextual detail in memories of perceived information. Johnson and Raye, 1981, Johnson and Raye, 2000 suggest that a decision about the internal or external nature of a memory is made based on a weighted combination of the active features during remembering, or via a matching process based on the characteristics of previous comparable memories. For example, if people hear some words from a speaker and imagine others, they are more likely later to mistakenly claim to have heard words that were only imagined, if their imagery was in the speaker's voice rather than in their own (Johnson, Foley, & Leach, 1988). According to the SMF, in addition to such relatively automatic heuristic attributions based on qualitative characteristics of mental experiences, reality monitoring (and source monitoring in general) also sometimes involves more deliberate/systematic processes that consider current experience in light of previous knowledge. For example, a ‘memory’ that is inconsistent with the report of someone else present at the time of an event might be doubted, whereas external ‘evidence’ (e.g., a train ticket) might increase confidence (Johnson, Suengas, Foley, & Raye, 1988).

An impairment in reality monitoring ability has been implicated in symptoms of mental illness and associated more widely with the occurrence of anomalous perceptions and false memories (Johnson, 1991, Johnson and Raye, 1998, McKay and Dennett, 2009, Radaelli et al., 2013, Turner et al., 2010). For example, auditory verbal hallucinations may arise from a failure to recognise the self-generated nature of inner speech (Frith, 1992, Frith and Done, 1988, Hoffman, 1986). Such a proposal is supported by observations that patients with schizophrenia exhibit behavioral deficits in reality monitoring, which tend to be observed even in the absence of deficits in recognition memory (Fisher et al., 2008, Keefe et al., 2002, Stephane et al., 2010, Szöke et al., 2009, Vinogradov et al., 1997, Vinogradov et al., 2008). Such findings suggest there may be overlapping decision processes for determining the internal or external source of information that underlie both memory-based reality monitoring and the reality testing of current perceptual experience. Further evidence supporting this link comes from the observation that patients with schizophrenia exhibit reduced brain activity during reality monitoring tasks within the medial anterior prefrontal cortex (Garrison, 2015, Vinogradov et al., 2008), a region associated with discriminating real from imagined information (Simons et al., 2006, Simons et al., 2008).

The processes involved in determining internal or external source during reality monitoring might apply not only to the origin of memories and real-time perceptual information, but also to discriminating the origin of knowledge, attitudes and beliefs (Johnson, 1988, Johnson, 1991, Slusher and Anderson, 1987). The observation of reality monitoring impairment in patients with schizophrenia who experience delusions (e.g., Thoresen et al., 2014) suggests that weakened reality monitoring may result in the establishment of a delusional belief through an initial hallucinatory false percept or unrecognised thought (Fletcher and Frith, 2009, Maher, 1974) and/or from failure of subsequent reasoning processes which supports the maintenance of the delusion (Turner & Coltheart, 2010), consistent with the SMF (Johnson, 1988, Johnson and Raye, 2000) and related two-factor theories of delusions (Coltheart, 2010). Reality monitoring impairment has been demonstrated in patients with anosognosia for hemiplegia compared to hemiplegic patients without anosognosia (Jenkinson, Edelstyn, Drakeford, & Ellis, 2009) suggesting a possible overlap between processes involved in monitoring action and perceptual information. Furthermore, a source monitoring explanation also accords with observations of reality monitoring impairment in individuals who experience false memories, such as patients with confabulations (Turner et al., 2010). Such individuals often exhibit temporal confusion (Schnider & Ptak, 1999) consistent with their failure to recognise an activated memory as pertaining to the past. A reality monitoring impairment during current thought or imagination might result in the experience of bizarre or fantastic confabulations, unrelated to reactivation of previous memory for previous events. Alternatively, spontaneous or provoked retrieval of a previous memory with insufficient source information might result in memory-based confabulations, with the error arising from the misattribution of mnemonic content to current experience.

An intriguing finding from the reality monitoring literature is that participants often exhibit an externalization bias as evidenced by a greater likelihood of falsely attributing new items to an external than internal source, or a greater proportion of imagined stimuli erroneously judged to have been perceived than perceived stimuli judged to have been imagined (Johnson, Raye, Foley, & Foley, 1981). There is much evidence for such an externalization bias in healthy individuals (Anderson, 1984, Foley et al., 1983, Hashtroudi et al., 1989, Hicks et al., 2002, Johnson et al., 1981) and in patients with mental illnesses such as schizophrenia (Bentall et al., 1991, Brébion et al., 2000, Brunelin et al., 2006, Seal et al., 1997, Waters et al., 2006, Woodward et al., 2007). Asymmetric source misattributions presumably reflect something about the evidence assessed and/or the criteria used in evaluating mental experience (Johnson et al., 1981, Marsh and Hicks, 1998). For example, a low threshold-level of perceptual information taken as evidence that information is external would produce externalization errors (Bentall & Slade, 1985). A belief that one would always remember generating an item (e.g., ‘remember’ cognitive operations information) would result in ‘memories’ without such information (e.g., false positives on new items) tending to be attributed to an external source (the ‘*it had to be you effect*’; Johnson et al., 1981, Johnson and Raye, 1981). A recent meta-analysis suggests that a tendency to misattribute internal events to external sources is associated with hallucinations in clinical participants and hallucination proneness in nonclinical participants (Brookwell, Bentall, & Varese, 2013).

Although the SMF incorporates the idea that self-generation may produce a variety of cues relating to source (e.g., records of central processes leading to the initial generation of a concept, motor components of speech production or writing, perceptual detail from one's voice or seeing what one has written, etc.), studies testing predictions of the SMF have tended to focus on the more central rather than peripheral components (e.g., Durso and Johnson, 1980, Finke et al., 1988; although see, Foley et al., 1983). A related idea that highlights the overt aspects of self-generation is that monitoring the origin of one's own actions might occur via processes that predict the associated sensory consequences and compare them with forward modelling or efference copy information (Feinberg, 1978). For example, a comparator model of motor control proposes that the central nervous system maintains internal representations of bodily states. One of these, the forward model, uses an efference copy (an internally generated duplicate produced through neuronal, or corollary, discharge) to predict the sensory consequence of motor commands whenever movement is initiated. According to this model, the matching of a top-down efference copy with the bottom-up sensory input provides subsequent awareness of the self-generated nature of the action (Miall and Wolpert, 1996, Seal et al., 2004).

This model can be applied to the cognitive operations information that the SMF proposes is used in the identification of the self-generated origin of thought and imagery. For example, corollary discharges from speech commands are considered ordinarily to prepare auditory cortex for self-generated speech (Feinberg, 1978, Jones and Fernyhough, 2007). Alien thoughts or hallucinations might arise through an impairment in either the generation of corollary discharges, or in the matching process itself, resulting in internally generated thoughts that are not perceived as having been originated by the self (Frith & Done, 1988). Notably, such deficit(s) would produce an external directionality of errors as, in the absence of an efference copy signal or a failure in the matching process, information would be assumed to be externally perceived. There is much evidence for abnormalities in the awareness of motor action in patients with schizophrenia (e.g., Blakemore et al., 2002, Frith et al., 2000, Posada et al., 2007), with the impairment also implicated in delusions of control where patients believe that their own actions are being influenced by an outside force (Frith & Done, 1989). Impaired self-monitoring has also been demonstrated in patients with anosognosia for hemiplegia who show poorer performance when compared with hemiplegic patients without anosognosia, on an internal source monitoring task involving the recollection of whether an action had previously been performed or imagined (Saj, Vocat, & Vuilleumier, 2014).

However, while evidence of impaired action-monitoring in schizophrenia and anosognosia is clear, the widespread implications of these deficits have been queried. In particular, theoretical arguments have been raised against the proposal that action-monitoring deficits lead to the generation of auditory hallucinations, with Gallagher (2004) questioning whether the generation of thought has the same physiological consequences as the generation of motor action. Furthermore, despite some reports of verbal self-monitoring deficits in patients with hallucinations (Brookwell et al., 2013), there is no direct evidence for a comparator system for auditory processing. Nevertheless, there is evidence that the more cognitive operations that are involved in generating words (Johnson et al., 1981) or mental images (Finke et al., 1988), the greater the accuracy of reality monitoring. Thus, the central idea that thought, speech, and other actions involve cognitive operations that generate cues in real time about the current origin of mental experience, and that persist as records that can be used later as cues in remembering, remains to be further explicated.

In short, a key proposition of the SMF is that reality monitoring involves assessment of the characteristic features of the information being reviewed. So for example, the amount and type of either perceptual information or of cognitive operations information activated during remembering should affect the accuracy of reality monitoring judgments. To investigate these factors, and their joint effects, we varied the conditions under which participants encoded pairs of associated words (e.g., *Laurel and Hardy*, *Bacon and Eggs*). The first item of each pair was always presented to the participant, with the second item either also presented (the ‘perceived’ condition), or with the participant cued with the first letter to enable them to self-generate the second item (‘imagined’ condition). Participants subsequently undertook a reality monitoring test in which they were shown the first item of each pair and asked whether the second item had been perceived, whether they had imagined it, or whether it was a new word.

Two aspects of the encoding conditions were varied. The *modality* of the presented stimuli was either visual or auditory and the *action* participants engaged in when generating the second item was either to silently verbalize the word-pair using inner speech (‘think’ condition) or to speak it aloud (‘speak’ condition). Of primary interest was the impact that these two factors had on source memory for externally-derived and internally-generated information. We hypothesized that since participants were instructed to covertly or overtly vocalize the words, the representation that participants generated would be more similar to what they perceived in the auditory than the visual condition and, hence, reality monitoring should be more difficult in the auditory modality. The effect of type of action was a more open question. Compared to covert vocalization, overt speaking should provide more motoric records and greater auditory feedback information (Price, 2012). If the speak condition results in less accurate reality monitoring than the think trials, it would suggest that the similar motoric and perceptual feedback on perceived and imagined trials from speaking reduces the discriminability of these two sources. If speaking produces better reality monitoring accuracy than thinking, it would suggest that whatever is added by speaking (e.g., motoric and/or perceptual information) is different when vocalizing information that has only been perceived compared to vocalizing information that has been generated. Finally, this design allowed us to investigate whether stimulus modality and type of action both affect externalization bias and whether their effects are independent or interact.

## 2 Methods

### 2.1 Participants

45 native speakers of English (13 male, 32 female) aged 18–35 (*M* = 21.2, SD = 2.7) took part in the experiment. All participants had normal or corrected to normal vision and hearing, had English as their first language and had lived in the UK for all or most of their lives (two had moved to the UK at the age of five).

### 2.2 Design and procedure

The reality monitoring task was administered using E-Prime 2.1 (Psychology Software Tools) software with responses made through the computer keyboard. Auditory stimuli were played through headphones worn by the participants for the duration of the experiment, with the sound adjusted to a comfortable level using practice stimuli before the task began. The task was adapted from one used previously (Simons et al., 2006, Simons et al., 2008) and involved the initial presentation of word-pairs followed by a test phase in which the participant was asked to indicate whether a word had earlier been presented within an intact word-pair using the response ‘*perceived*’, or had been presented in a word-pair which had needed to be completed by imagining the missing word, with the response ‘*imagined*’. Previously unseen words were also used in the test phase, requiring a ‘*new*’ response. The stimuli consisted of 288 well-known word-pairs (e.g., ‘Laurel and Hardy’, ‘Bacon and Eggs’), which had been pilot tested to ensure their familiarity among adults in the target age range, with a culturally English background. The task comprised eight separate study and test blocks, with 24 word-pair stimuli in the study phase and an additional 12 ‘new’ words included in the test phase.

The experiment used a within participants design involving the manipulation of three factors: action (speak or think), modality (visual or auditory) and the source condition (perceived or imagined). In visual trials, the study phase word-pairs were presented on the computer screen with no auditory input. In auditory trials, the study phase word-pairs were provided through the headphones with no visual input. Following presentation of the word-pairs, either visually or aurally, and in either perceived or imagined trials, the participants were then instructed to either speak the completed word-pair aloud, or to verbalise it internally (i.e., to ‘think’ it). Two blocks were used for each of the four different combinations of the action and modality factors to provide study phase trials that comprised all combinations of auditory/speak, auditory/think, visual/speak or visual/think.

**Fig. 1:**
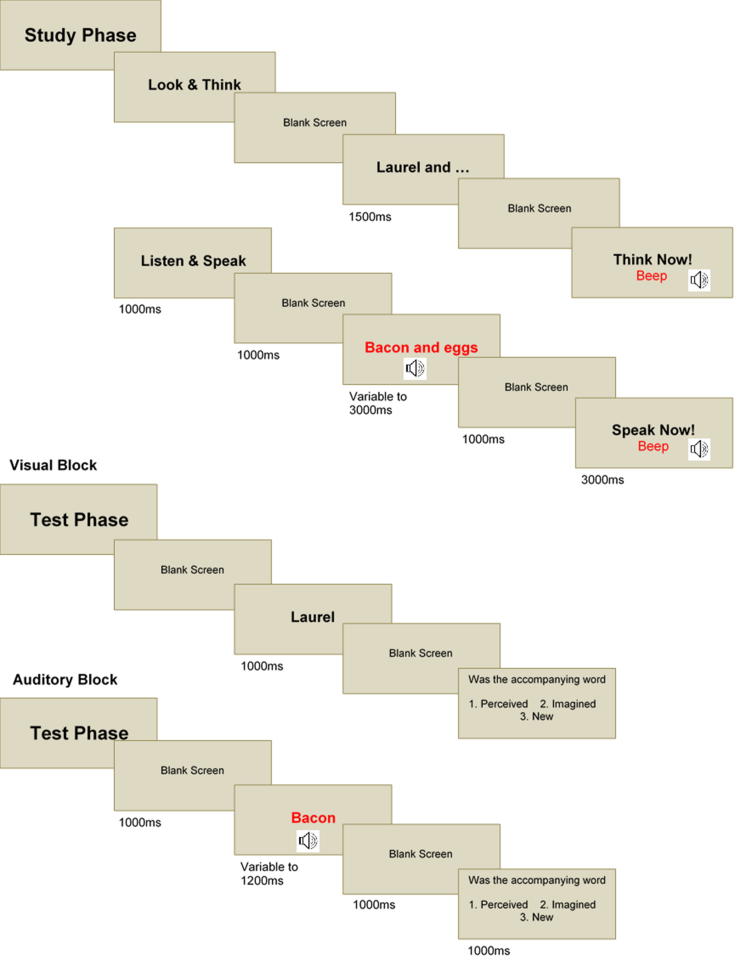
Examples of task stimuli. Note: red text indicates auditory presentation. Three different voices were used to record the word-pairs to ensure variety, and the task was fully counterbalanced across participants for the use of voices, the presentation of word-pairs as perceived, imagined and new, and for the order of the eight blocks (two per four study phase conditions).

The timings and stimuli used in the task are shown in Fig. 1. Each block of the task commenced with a display screen indicating the relevant conditions for that block: ‘Look and Think’, ‘Look and Speak’, ‘Listen and Think’ or ‘Listen and Speak’ to orient the participant to the study condition associated with that block of 24 stimuli. A word-pair was then presented aurally (3000 msec max) or visually (1500 msec [approximating the mean time taken in the auditory condition]) with ‘imagined’ visual word-pairs having the second word substituted by three dots (‘Laurel and …’) and aurally by a silence (‘Laurel and ’). A blank screen/silence was then followed by an audible ‘beep’ and a visual display screen instructing participants either to ‘Think Now’ or ‘Speak Now’. This procedure was repeated for each of the 24 word-pairs in that study phase block, each trial separated by a second blank screen or silence.

A study phase block was followed immediately by its corresponding test phase. The first word of a studied word-pair, or a new word, was presented aurally (mean presentation 800 msec) or visually (1000 msec) consistent with the modality condition of the study phase. After a blank screen/silence (1000 msec) a test screen was presented with the question: ‘*Was the accompanying word 1. Perceived, 2. Imagined, 3, New?*’ The participant had a maximum of 4 s to respond with the numbered response but the task was self-paced (no responses were made outside this response window). This procedure was repeated for each of the 36 words in that test phase block, each trial separated by a further blank screen/silence. The order of word-pairs presented in both study and test phases was pseudo-randomised such that there was no sequence of perceived, imagined or new words greater than three items in length. Each block of the task lasted for around 6 min and the eight blocks (two per condition) were run sequentially without a break.

### 2.3 Data analysis

Arcsine data transformations were used to enable the normality assumptions of parametric tests to be met (Howell, 2012). Thereafter, old/new recognition accuracy was calculated as the adjusted hit rate (i.e., hits – false alarms, where ‘hits’ were defined as the proportion of correct responses to items recognised as previously presented (‘old’), and ‘false alarms’ as the proportion of newly presented items which were incorrectly identified as old). Reality monitoring accuracy was calculated as accurate source responses divided by correct responses recognising an item as old.

Conditional misattribution errors were calculated for perceived and imagined trials as the number of responses made for the alternative reality monitoring response as a proportion of total errors made. So for example, ‘Imagined judged Perceived’ errors were calculated as the number of ‘Perceived’ responses divided by the sum of ‘Perceived’ and ‘New’ responses that were made to imagined trials. This gives a measure of misattribution error unrelated to overall accuracy for each condition. 12 participants made no errors for one or more of the study conditions and were excluded from the misattribution analysis of variance, leaving 33 participants for that analysis. Note that including all participants' data and comparing imagined judged perceived and perceived judged imagined errors across the four conditions showed the same overall externalization bias (*t* > 2.72, *p* < .01) as reported below.

Preliminary analyses confirmed the absence of significant effects of potentially confounding variables on old/new recognition, reality monitoring accuracy or error rates, of participants' age, sex or handedness, or of the voice used for the auditory condition. Thus, these variables are not discussed further.

## 3 Results

### 3.1 Old/new recognition

The effect of action (speak, think), modality (visual, auditory) and word-pair source (perceived, imagined) on old/new recognition was assessed first (Table 1). Analysis of variance indicated a significant main effect of source, *F*(1, 44) = 72.199, *p* < .001, *η*_p_^2^ = .621 and action, *F*(1, 44) = 32.857, *p* < .001, *η*_p_^2^ = .428, but no significant effect of modality, *F*(1, 44) = 1.450, *p* = .235, *η*_p_^2^ = .032. There was also a significant interaction between source and modality, *F*(1, 44) = 41.872, *p* < .001, *η*_p_^2^ = .488, but no other interactions were significant, *F*(1, 44) < .321, *p* > .574, *η*_p_^2^ < .007.

In short, recognition memory was better for imagined stimuli (*M* = 89.8%, SD = 6.8%) compared with those that had been perceived (*M* = 82.8%, SD = 7.7%), a type of generation effect (Bertsch, Pesta, Wiscott, & McDaniel, 2007). Recognition memory was also enhanced by the act of speaking (*M* = 89.4%, SD = 5.9%) compared with thinking at the point of encoding (*M* = 83.2%, SD = 8.9%), a type of production effect (MacLeod, Gopie, Hourihan, Neary, & Ozubko, 2010). The modality of presentation also had an effect on recognition memory, but this differed for perceived and imagined stimuli: recognition memory was better with auditory than visual presentation for perceived stimuli: *t*(44) = 2.590, *p* = .013, *d* = .393, but better with visual than auditory presentation for imagined stimuli: *t*(44) = 4.134, *p* < .001, *d* = .635. This interaction likely reflects that recognition in this task is based not simply on undifferentiated familiarity, but by the more specific features participants are assessing in making source attributions. Our main analyses of reality monitoring (see below) were conditionalized on old/new recognition and more specifically address questions about conditions that affect judgments about the origin of memories.

### 3.2 Reality monitoring

**Fig. 2:**
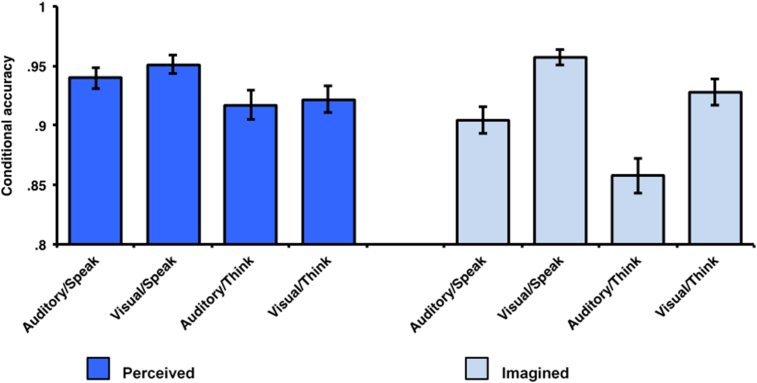
Reality monitoring accuracy for perceived and imagined trials. Note: Error bars represent ±standard error of the mean.

A 2 (action: speak, think) × 2 (modality: visual, auditory) × 2 (source: perceived, imagined) repeated measures ANOVA (Fig. 2) revealed significant main effects of action, *F*(1, 44) = 22.408, *p* < .001, *η*_p_^2^ = .337, modality *F*(1, 44) = 23.810, *p* < .001, *η*_p_^2^ = .351, and source, *F*(1, 44) = 5.341, *p* = .026, *η*_p_^2^ = .108. Furthermore there was a significant interaction between modality and source, *F*(1, 44) = 15.120, *p* < .001, *η*_p_^2^ = .256, but no other interactions were significant: *F*(1, 44) < .823, *p* > .369, *η*_p_^2^ < .018.

The main effect of action reflects that source accuracy was greater for speak than think trials. The modality by source interaction reflects that, for perceived trials, there was no significant difference between aurally and visually presented word-pairs [*t*(44) = .618, *p* = .540, *d* = .099] but, for imagined trials, there was significantly lower accuracy for aurally presented word-pairs compared with visually presented word-pairs, *t*(44) = 6.752, *p* < .001, *d* = 1.107.

Thus, reality monitoring performance was better for spoken than thought items, and auditory presentation resulted in poorer reality monitoring performance than visual presentation, especially for the self-generated items.

### 3.3 Misattribution errors

**Fig. 3:**
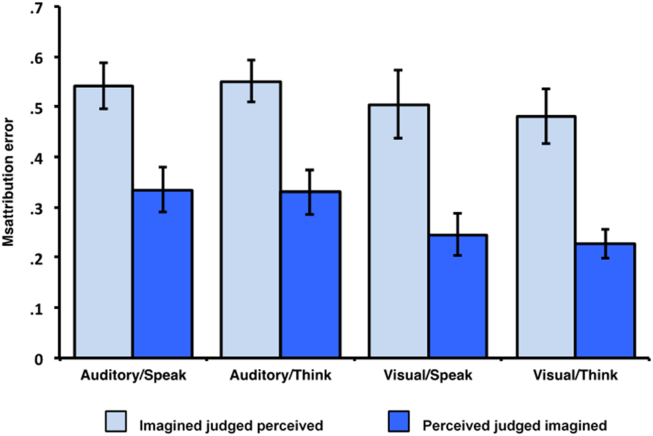
Misattribution errors.

Conditional misattribution error rate was calculated as a measure independent of reality monitoring accuracy to give an indication of the proportion of errors that were misattributed to the alternative reality monitoring condition. The error rates for misattributions of perceived and imagined stimuli for the different action and modality conditions are shown in Fig. 3. There was a significant main effect of error direction *F*(1, 32) = 21.606, *p* < .001, *η*_p_^2^ = .403, indicating that the proportion of externalization errors (‘Imagined judged Perceived’) was greater than internalization errors (‘Perceived judged Imagined’). There was no significant main effect of action, *F*(1, 32) = .051, *p* = .822, *η*_p_^2^ = .002, or modality, *F*(1, 32) = 3.066, *p* = .090, *η*_p_^2^ = .087, and no significant interactions, *F*(1, 32) < .261, *p* > .613, *η*_p_^2^ < .008.

## 4 Discussion

In the present experiment, participants were presented with intact word-pairs on some trials and, on other trials, they completed word-pairs by imagining the second item when cued with the first. We investigated the effect of manipulating at encoding the perceptual modality (visual *vs* auditory) in which stimuli were presented and the action of the participant (speak *vs* think) during encoding on subsequent reality monitoring (‘did you perceive or imagine the item associated with this cue?’). We observed poorer reality monitoring in the think versus speak conditions, and an effect of presentation modality, in which accuracy was lower for imagined, but not perceived, trials that had been presented auditorily compared to visually. Participants were more likely to misattribute imagined items to perception than perceived items to imagination (i.e., higher rate of externalization than internalization errors); moreover, this asymmetrical error pattern was unaffected by whether stimuli were presented visually or aurally and whether participants spoke or only thought their responses during encoding. Below we discuss these findings in light of the SMF and with respect to their potential relation to hallucinations, confabulation and delusions.

### 4.1 The effect of modality

According to the SMF account, reality monitoring accuracy should be better the greater the difference between the perceptual content of external and internal stimuli. Thus, we expected that reality monitoring accuracy would be higher for visually presented compared to aurally presented stimuli. That is, it should be easier to discriminate later between perceived visual words and inner speech than between perceived auditory speech and inner speech because self-generated inner speech is more likely to have auditory than visual qualities. Reality monitoring was better in the visual than the auditory condition, but only for imagined stimuli. Note that this was not because of lower old/new recognition for imagined that perceived items in the auditory conditions (see Table 1). Thus, this dissociation between old/new recognition and source monitoring is consistent with the idea that imagined items were particularly difficult to identify as self-generated in the auditory condition not because they gave rise to ‘weak’ memories but because the specific characteristic(s) either encoded or assessed during later reality monitoring were not as reliable cue(s) to source, presumably because they seemed more like perceptually-derived memories in their auditory qualities.

The observed findings invite a speculative account of the greater prevalence of auditory verbal hallucinations compared to visual hallucinations in patients with schizophrenia and other clinical conditions (Aleman & Larøi, 2008). Reality monitoring of visual stimuli may, on average, be easier compared to auditory stimuli. That is, substantially greater perceptual content of perceived visual stimuli compared to the level of visual detail in imagined visual stimuli may make it relatively easy to distinguish external from self-generated visual imagery, whereas the source-related detail available in inner speech may typically be more similar to that of external speech. Of course, individual differences in visual and auditory imagery should modulate such effects, and indeed our data hint at this with only a moderate correlation found in participants' reality monitoring accuracy for imagined stimuli presented in the auditory and visual modalities. Consistent with such predictions, there is mounting evidence that auditory and visual hallucinations may be associated with the sporadic over-stimulation of sensory association cortices, such as voice selective regions in the superior temporal gyrus for auditory hallucinations and the visual cortices for visual hallucinations (Allen, Larøi, McGuire, & Aleman, 2008), which might produce more vivid perceptual content in comparison to that typically associated with self-generated information. Evidence to support this proposal comes from fMRI analysis of cortical activation during state studies of both auditory and visual hallucinations (Garrison, 2015, Kühn and Gallinat, 2012), which have identified hallucination-related activation in the respective sensory cortices, and from research observing neural activity in speech sensitive auditory cortex even during silence (Hunter et al., 2006). Converging evidence comes from fMRI studies of healthy participants. Similar brain regions are active during visual perception and imagination (Johnson and Johnson, 2014, Johnson et al., 2007, O'Craven and Kanwisher, 2000) and scores on a scale measuring proneness to hallucinations were related to activity in superior temporal gyrus for misattributions of imagined to perceived spoken words during a reality monitoring test (Sugimori, Mitchell, Raye, Greene, & Johnson, 2014).

### 4.2 The effect of action

Also of interest is that speaking compared to thinking during encoding resulted in an advantage later in reality monitoring. Both thinking and speaking include critical cognitive operations that generate the target response on imagination trials – cognitive operations information that later could provide cues about the origin of remembered items. Beyond thought, speaking includes further action planning and execution and additional perceptual detail based on auditory feedback from the participant's voice (Price, 2012). If participants had spoken *only* the imagined items, this additional information could provide potential information for making source attributions. However, during encoding, the perceived items were also spoken by the participant, so why would speaking confer a reality monitoring advantage over thinking in this context? In the Introduction we suggested that repeating aloud something that was only perceived might be different than speaking aloud something that had been generated. One possibility is that “compiling” a plan for speaking a word that has just been perceptually presented is more automatic and/or less complex than compiling a plan for speaking a word that has not just been presented but, rather, has just been generated. Furthermore, if plans for overt speaking are more complex than plans for covert thinking, it seems reasonable that the difference in the planning operations generated between imagined and perceived trials would be greater in the speak than the think condition. If so, the combined cognitive operations involved in generation and response planning should better differentiate imagined from perceived items in the speak than the think conditions. Such an idea that speech involves more complex self-monitoring than thought could be easily represented in comparator forward models of action planning as highlighted in the Introduction (Feinberg, 1978, Jones and Fernyhough, 2007).

In any event, the fact that speaking did not reduce reality monitoring compared to thinking suggests that the speak condition may be especially appropriate for studying reality monitoring in patient populations in which compliance on think trials may be an issue. It is also notable that the cognitive operations associated with generating imagined responses, plus any possible additional useful records generated by speaking (e.g., action planning/execution processes) did not interact with the advantage derived from visual compared to auditory presentation. This suggests that records of perceptual detail and operations involved in self-generation make independent contributions to reality monitoring in healthy adults in this task.

### 4.3 Asymmetry in reality monitoring errors (externalization bias)

Previous observations have suggested that participants in reality monitoring studies often exhibit a greater proportion of external than internal misattribution errors (Johnson et al., 1981, Johnson and Raye, 1981). This tendency to make disproportionate numbers of externalization errors was also observed in the current experiment. Furthermore, the level of misattribution bias was independent of both the modality of presentation of the stimuli, and of the action of the participant during encoding. This suggests that a common factor may affect all of the conditions in the study, for example, the quality of the cognitive operations evidence. Poor or absent cognitive operations information regarding the initial generation of responses on imagine trials would increase the chances of an externalization error. The stability of the externalization bias across the conditions in this study is also consistent with evaluation of evidence prior to efference copy/sensory matching in a self-monitoring process related to comparator models for inner speech (Feinberg, 1978, Jones and Fernyhough, 2007). The current study cannot distinguish between these alternatives. However, what is clear is that the directionality of bias is consistent with the nature of perceptual errors in hallucinations and non-memory-based confabulations, where internally generated information tends to be ascribed to an external source (e.g., inner speech is recognised as a spoken voice) much more often than an external perception is misrepresented as imagery or thought.

Hallucinations, confabulation and delusions represent a broad range of clinically significant subjective experiences that involve source discrimination failures (e.g., Johnson, 1988). Combining ideas from current theoretical models of on-line reality discrimination with ideas from the SMF should advance a broad theoretical framework for such experiences. For example, an impaired self-monitoring process which attributes ongoing, internally generated information to that of an external agent (consistent with efference copy accounts of motor control) could help explain the mechanism by which self-generated cognitions may come to be experienced as alien (Jones and Fernyhough, 2007, Waters et al., 2012; but see, Gallagher, 2004). The impaired self-monitoring of internally generated imagery might, for instance, lead to the development of a fantastic confabulation as the information acquires the phenomenal qualities of being related to an external source. This broader account of reality discrimination is also consistent with theoretical explanations relating to impairments in the recognition of action in anosognosia (Fotopoulou, 2010, Fotopoulou et al., 2008, Saj et al., 2014) as well as in delusions of control (Frith & Done, 1989).

Other key issues for investigation include the involvement of underlying brain regions, particularly medial prefrontal cortex and anterior cingulate cortex, which are implicated in monitoring processes (Buda et al., 2011, Garrison et al., 2015, Simons et al., 2008, Simons et al., 2006, Vinogradov et al., 2008). Further understanding is also needed of the inter-relation between faster non-deliberate judgement processes which may operate during perception or during remembering on initially activated information, and slower more deliberative metacognitive evaluation which might take into account existing beliefs and knowledge and which might explain the embellishment and/or continued maintenance of impaired beliefs. Investigating such questions related to both the mechanisms of reality discriminations and their neural substrates, could thus provide both cognitive insight into how we discriminate self-generated information from that which is real, as well as routes towards the development of therapeutic interventions for symptoms of mental illness that reflect failures in reality monitoring.

## Figures and Tables

**Table 1:** Old/new recognition accuracy for perceived and imagined trials.

	Perceived		Imagined	
	Mean	SD	Mean	SD
	%	%	%	%
Auditory/speak	87.6	9.6	90.8	8.3
Visual/speak	84.9	9.0	94.2	6.1
Auditory/think	80.8	13.4	83.9	11.9
Visual/think	77.8	11.9	90.4	8.1
